# Differentiated approach to Chêneau brace choice for the scoliosis treatment

**DOI:** 10.1186/1748-7161-7-S1-O32

**Published:** 2012-01-27

**Authors:** D Chekryzhev, A Mezentsev, D Petrenko

**Affiliations:** 1 Orthospine Ltd, Kharkiv, Ukraine; 2 Sytenko Institute of Spine and Joint Pathology, Kharkiv, Ukraine

## Background and purpose

To validate choice of the Cheneau brace according to scoliotic deformity type.

## Materials and methods

Study group included 1700 patients with AIS treated by Cheneau brace [1]. Major deformity Cobb angle ranged 20°-40°, spinal rotation ranged 15°-35°. Age of the patients was ranged 11-15 years old. Radilogic mesurements and clinical presentation of the spinal deformity during the investigation were assesed [2]. Mean follow-up was 5 years. In this study Rigo's clinical and radiological criteria [3] and SRS terminology were used.

## Results

According to obtained data four scoliotic deformity patterns were defined: primary thoracic and secondary lumbar/thoracolambar curve (pattern 1); primary thoracolumbar and secondary lumbar curve (pattern 2); primary lumbar curve with/without secondary thoracic curve (pattern 3); triple curve deformity (pattern 4). This classification allowed to deifine following 6 brace types : for 1 pattern - braces of Tl, T2, T3 types. For 2 pattern - brace T3 type; for 3 pattern - braces of L1, L2, Tl and T2 types. For 4 type pattern - brace of CTL type.

## Conclusions

Scoliosis deformity classification provides differentiated Cheneua brace selection according to the deformity pattern.
